# Determinants of Persistent Underweight among Children, Aged 6–35 Months, after Huge Economic Development and Improvements in Health Services in Oman

**Published:** 2007-09

**Authors:** Deena Alasfoor, Pierre Traissac, Agnès Gartner, Francis Delpeuch

**Affiliations:** 1 Department of Nutrition, Ministry of Health, Sultanate of Oman; 2 Nutrition Unit, **Institut de Recherche pour le** Développement (**IRD**) (**WHO Collaborating Centre for Nutrition**), **B.P. 64501, Montpellier, France**

**Keywords:** Birth-weight, Case-control studies, Childcare, Child nutrition disorders, Underweight, Oman

## Abstract

Over the last decades, health indicators have witnessed major improvements in the Sultanate of Oman. This study was aimed at factors associated with underweight among children in four regions of Oman, as, in 1998, underweight was prevalent among 17.9% of children aged less than five years. A case-control study was conducted in 2002: 190 cases were 6-35-month old children with weight-for-age <−2 z-scores. Controls were individually matched by village of residence, sex, and age. The questionnaire included anthropometry of children, child-feeding practices, morbidity, anthropometry of mothers, parity, birth-spacing, and socioeconomic characteristics. Conditional logistic regression was used for analyses. Birth-weight of <2,500 g was strongly associated with underweight and also were height of mother, low level of education of mother, bad quality of water in households, diarrhoea of children in the last two weeks, and regular use of infant formula. Factors, such as birth-weight, height of mother, supply of safe water in household, and care for mothers and children were the determinants of persistent underweight after huge economic development and improvements in health services. Further research is also needed to investigate further specific determinants of low birth-weight in the Omani context and try to disentangle emaciation and determinants of linear growth retardation.

## INTRODUCTION

In the last three decades, due to oil revenues and policy choices, the Sultanate of Oman has undergone drastic economic and sociodemographic changes. Between 1975 and 2003, its human development status, as assessed by the human development index (HDI), experienced the world's largest observed increase for this period, from 0.493 to 0.780 (1). Oman now ranks 71 among 177 nations in the first quarter of the medium human development countries. In the same period, most health indicators witnessed major improvements (2). Between 1960 and 2002, the rate of infant mortality dropped from 164% to 11%, and the rate of mortality of children aged less than five years (under-five mortality) went down from 280% to 13% (3). Rate of infant immunization rose from 10% in 1980 to 99% in 2001 (4). Also, during the same period, the prevalence of different forms of undernutrition decreased markedly. Between 1980 and 1998, underweight among Omani children aged less than five years decreased from 62.9% to 17.9% and stunting from 20.3% to 10.4% (5,6). Wasting decreased from 12.8% in 1995 to 7.2% in 1998 (6). By the international standards of the World Health Organization (WHO) for prevalence of child malnutrition (7), level of underweight in Oman is still in the ‘medium’ range, even if the health of all Omani children can be monitored due to sufficient resources and adequate heath services.

The conceptual framework of our analysis is that of the international conceptual framework of the causes of malnutrition (8). Due to the specific Omani context, our hypotheses were that (a) the factors from the ‘health environment and services’ (with the exception of water supply which is historically important in Oman) and ‘household food security’ categories of the underlying causes of malnutrition (8) could be considered at a sufficient level (9) and (b) that risk factors of underweight could, thus, be more likely found in the child and maternal care categories (also without excluding the legacy of the predevelopment years via prenatal factors). Despite a few descriptive studies (10,11) or aimed at specific risk factors (10,12), no data were available to assess the relationships between a sufficiently large number of factors and the anthropometric status of children. Therefore, this study was aimed at assessing the risk factors of underweight among young Omani children. For that purpose, a matched case-control study was conducted among 6–35-month old children residing in four regions of Oman.

## MATERIALS AND METHODS

### Study design

A 1:1 case-control study with individual matching was deemed to be the most efficient for the study (13,14). Cases were defined as underweight children aged 6–35 months. Controls were non-underweight children matched by village of residence, sex, and age (within one month).

### Study sample

For a 0.05 first-type error risk and a power of 0.80, assuming a 20% prevalence of exposure among controls, the sample size to detect an odds ratio of 2.0 was computed as 187 case-control pairs (n=374) (calculation performed with the Epitable module in the Epi Info software, version 6.04 (15)).

Cases were selected from four (Muscat, Dhakhilia, North and South Sharqia) of the 10 health regions in Oman which represent different situations and prevalences of underweight among children aged 0–59 month(s) (respectively 12.8%, 22.7%, 26.8%, and 19.7% in 1998) (6).

### Inclusion of case-control pairs

In each commune, the Community Support Group members screened all children aged less than three years for cases and suitable controls using the reference growth charts and individual screening forms, including relevant inclusion/exclusion information. After verification at the Department of Nutrition of the Ministry of Health in Muscat, the case-control pairs were included based on age, validation of weight-for-age status (by calculation by computer), absence of any visible congenital diseases that might affect growth, and accuracy of the matching criteria, as reported by the field teams. All participant mothers gave their free and informed verbal consents.

### Variables

The questionnaire featured items relating to the child himself/herself, his/her mother, father, and the household that are usually assumed to be potentially linked to malnutrition of the child. Moreover, consanguinity of parents was also considered as it is frequent in Oman (11) and could have an effect on the health of the child.

Anthropometric measurements were made under standardized conditions according to the recommendations of WHO (7). Weight of the child was measured with Uniscale weighing scales (with 100 g precision) (Seca, Birmingham, UK). Calculation of age was based on the date of birth of the child verified on an official health document (which was also used for collection of birth-weight data). Using the Epinut module of the Epi Info software (version 6.04) (15), weight-for-age was computed for each child and expressed in z-scores (WAZ) of the international reference values (16). Underweight was defined as WAZ <−2 z-scores (7). Body mass index (BMI=weight/height^2^) was used for assessing the nutritional status of the mother, <18.5 kg/m^2^ and ≥25 kg/m^2^ defining underweight and overweight respectively (7).

Haemoglobin (Hb) concentration for the child and the mother was measured (HemoCue AB, Angelholm, Sweden), and Hb status was described using 11 and 12 g/dL cut-off for the child and for the mother respectively (17).

The current breastfeeding status of the child was considered adequate if breastfed and age was less than 24 months or not breastfed and age was ≥24 months, inadequate otherwise. Complementary feeding was considered timely if age of the child at start was 6–8 months. A child diversity index was computed from 24-hour recall data using four food groups: cereals, vegetables/fruits, dairy products, and proteins.

A household water-quality index was derived from source of water and method of delivery and storage of water: good for water from pipes directly from well or traditional rural irrigation system ‘*falaj*’, average if water from wells, *falaj*, or desalination plant and delivered by vehicle, and ‘bad’ otherwise. A household economic level proxy was computed by correspondence analysis from type of house, ownership of house, presence of servants, number of persons per room, presence of electricity, refrigerator, computer, and Internet, and number of cars. The first principal component displaying a gradient of household ‘wealth’ was used as a summary index of household wealth. Households were grouped in ‘low’, ‘medium’, and ‘high’ levels according to terciles of this index (18). Based on the Ministry of National Economy methodology and a minimum necessary income of 30 Omani rials per person per month (19), household income ‘poverty gap’ was computed as total declared monthly income - (30 × number of household members). A household dietary diversity index (20) was computed based on usual consumption at least once a day of five food groups: cereals, vegetables, fruits, dairy products, and proteins. After the standardization of measurements and implementation of the questionnaire, actual data collection took place simultaneously in the four regions from May to August 2002.

### Data management and statistical analysis

Data-entry tools, including quality-checks, were developed using the data-entry module of the Epi Info software (version 6.04) (15,21). Data cleaning, management, and computation of derived variables and scores were performed with the Epi Info software and the SAS software (version 8.2) (22).

Statistical analyses were performed to take into account the 1:1 case-control design with individual matching on sex, age, and village of residence. To assess the differences between cases and controls, univariate conditional logistic regressions (13,14) with underweight (yes/no) as the response variable were used, the qualitative explanatory variables being considered one at a time. These univariate regressions were fitted to assess ‘unadjusted’ effects by likelihood ratio tests and to obtain odds ratios (ORs) using a maximum available sample size for each variable. To account for confounders and/or intermediate factors, variables significantly related to underweight at ∞=0.10 level and/or deemed of importance in the Omani context were retained for inclusion in a multivariate model. This latter analysis enabled us to assess the ‘adjusted’ effects (likelihood ratio tests) and to obtain ORs adjusted for all variables included in the model, based on the pairs for which values of all the retained variables were available (‘complete case analysis’ subsample). The conditional logistic regression models were fitted using PROC PHREG in the SAS software (version 8.2) (23). Unless stated otherwise, confidence intervals (CIs) were computed for 0.95 confidence level.

## RESULTS

Screening of all children in 53 villages resulted in 190 pairs (47, 44, 47, and 52 for the Muscat, Dhalhilia, North Sharqia, and South Sharqia regions respectively). For cases and controls, respectively, the mean WAZ was −2.42 (0.42) z-score and −1.01 (0.69) z-score, and the mean age was 24.1 (7.9) months and 23.8 (8.0) months (paired differences 0.95 CI −0.06–0.71). 50.5% of the children were boys.

### Univariate analysis of associations between underweight and potential risk factors

The results of univariate analyses showed that underweight was linked to variables from the different groups: prenatal factors, current health of the child, child-feeding practices, and socioeconomic status, but not with factors describing the health status of the mother (percentages are given in Table 1, relevant ORs and CIs quoted in the text).

**Table 1:** Univariate analysis of associations between underweight and potential risk factors

Variable	No.∗	Cases (%)	p value	Controls (%)
Prenatal factors				
Age (years) of mothers at birth	378		0.52	
<23		33.3		29.1
23–28		32.8		38.1
≥29		33.9	32.8	
Height (cm) (terciles) of mothers	374		0.0003	
<151		37.4		26.2
151–155		37.4		28.9
≥156		25.2		44.9
Time (months) elapsed since previous child	368		0.78	
No previous child		10.9		12.0
<24		26.1		23.4
≥24		63.0		64.7
Consanguinity of parents	380		0.79	
First-degree relatives		37.4		36.8
Second-degree relatives		22.1		24.8
None		40.5		38.4
Iron supplementation for mothers	380		0.024	
Yes		86.9		80.0
Birth-weight		376		<0.0001
<2,500 g		18.1		5.3
Health status of mothers				
Number of pregnancies	380		0.32	
≤2		17.4		23.7
3–5		33.1		29.5
≥6		49.5		46.8
Mother is pregnant		380	0.60	
Yes		22.1	24.2	
Body mass index (if not pregnant)	228		0.49	
<18.50 kg/m2		9.7		9.7
18.50–24.99 kg/m2		56.1		49.1
≥25.00 kg/m2		34.2		41.2
Haemoglobin status of mother	374		0.66	
<11g/dL if pregnant, <12 g/dL if not		58.8	56.7	
Child health				
Current haemoglobin status	378		0.71	
Hb <11 g/L		75.1		75.7
Child had diarrhoea in the last 2 weeks	380		0.12	
Yes		15.3		10.0
Child had ARI in the last 2 weeks	380		0.62	
Yes		22.6		24.8
Health regularly monitored	372		<0.0001	
Yes		74.2		50.5
Child-feeding practices				
Child adequately breastfed	378		1.00	
Yes		73.0		73.0
Mother usually stops breastfeeding at	380		0.40x	
≤12 months		20.0		22.1
13–18 months		22.6		26.8
>18 months		57.4		51.1
Child was ever given formula regularly	378		0.10	
Yes		30.1		22.8
Child given complementary feeding timely	380		0.32	
Yes		3.2		1.6
Child eats from separate plate	380		0.019	
Yes		49.0		38.4
Child dietary diversity index	378		0.31	
≤2		11.6		16.4
3		34.4		31.2
4		53.4		52.4
Socioeconomic status and environment				
Level of schooling of mothers	370		0.045
None		38.4		30.8
Primary		30.3		24.9
Elementary		11.9		15.7
Mother has professional occupation	380		0.015	
Yes		2.1		7.4
Marital status of mothers	380		0.65	
Married vs widowed, divorced		99.0		98.4
Level of schooling of fathers	376		0.44	
None		17.6		13.8
Primary		28.2		23.4
Elementary		22.3		26.6
Secondary and more		31.9		36.2
Professional occupation of fathers	376		0.14	
Manager, engineer, technician		35.1		30.9
Clerk, employee		20.7		13.9
Worker, labourer		44.2		55.2
Father lives in household	380		0.45	
Yes		76.8		73.7
Father is head of household	376		0.095	
Yes		78.7		85.1
Household size (persons)	380		0.92	
1–7		25.8		27.4
8–12		43.7		43.1
13 and more		30.5		29.5
Household water-quality index	380		0.13	
Bad		37.4		30.5
Average		36.8		42.1
Good		25.8		27.4
Household economic level proxy (terciles)	380		0.97	
First tercile: ‘low level’		34.2		33.2
Second tercile: ‘medium level’		32.1		32.6
Third tercile: ‘high level’		33.7		34.2
Total declared monthly income (rials) of household	380		0.035	
<250		33.2		24.2
250–499		36.8		37.9
500 and more		30.0		37.9
Household income: ‘poverty gap’	380		0.003	
<−100		24.7		16.8
−100 to +299		56.8		53.7
+300 and more		18.4		29.5
Household food-purchase decision	380		0.21	
Mother decides, she or father buys		70.0		75.3
Household dietary diversity index	380		0.38
≤2		19.0		16.3
3,4		54.2		60.5
5		26.8		23.2

∗ Maximum available sample size for each variable; ARI=Acute respiratory infection

### Health status of mothers and prenatal factors

The children of the shortest (respectively intermediate-sized) mothers were more at risk (OR=2.6, CI 1.5–4.4) (respectively OR=2.3, CI 1.4–3.8) of underweight than children of the tallest mothers. Children with low birth-weight (<2,500 g) showed an increased risk of underweight (OR=5.8, CI 2.2–15.0) when compared with normal children. Children born from mothers having received iron supplementation during pregnancy were more frequent among cases vs control. No risk of underweight was associated with age of mothers at birth, time elapsed since previous child, consanguinity of parents, number of pregnancies, mother pregnant or not, and BMI and Hb status of mothers.

### Child health and feeding practices

Children who had diarrhoea (vs not) in the last two weeks were marginally more at risk of underweight (OR=1.6, CI 0.9–3.0). Children whose health was regularly monitored were more numerous among cases than controls. No risk of underweight was associated with a recent episode of acute respiratory infection or with the current Hb status.

A child eating from a separate plate (vs not) was more at risk of underweight (OR=1.7, CI 1.1–2.8). If the child was ever given formula regularly (vs not) marginally increased the risk of underweight (OR=1.5, CI 0.9–2.3). Underweight was not linked to adequate breastfeeding, age when mother usually stops breastfeeding, the fact that the child's complementary feeding was timely, and the dietary diversity.

### Socioeconomic status and environment

Children of mothers with no schooling (OR=2.1, CI 1.1–3.7) or primary school (OR=2.0, CI 1.1–3.9) were more at a risk of underweight compared to those whose mothers had secondary education or more. Children whose mothers were engaged in a professional occupation showed a decreased risk of underweight vs children of non-working mothers (OR=0.3, CI 0.1–0.9). Professional occupation of the father was marginally associated with underweight. If the father was head of the household (vs not), it had a beneficial impact on the weight status of the child (OR=0.6, CI 0.4–1.1). Underweight was not linked to the marital status of the mother, the schooling level of the father, and if the father lived in the household or not.

Income of the household and poverty gap (the variable retained for the multivariate model) were linked to underweight. For the latter, the children from the poorer households (OR=3.6, CI 1.6–7.9) and the intermediate ones (OR=2.2, CI 1.2–4.1) had an increased risk of underweight when compared with households in the higher-income category. The quality of water in the household was marginally associated with underweight, with a risk of underweight increased for children from households with ‘bad’ quality of water vs ‘average’ or ‘good’ (OR=1.9 CI 1.0–3.5). Underweight was not associated with size, economic level, and the dietary diversity of the household, nor if mother (vs father) decided for purchase of foods.

### Multivariate analysis of associations between underweight and potential risk factors

The final multivariate analysis was performed on the ‘complete case analysis’ of the subsample (n=354). For comparison purposes (Table 2), unadjusted effects and ORs were also computed for each factor on this (n=354) subsample to assess that the differences stemmed only from the adjustment process and not from some sort of selection bias linked to missing values.

**Table 2:** Multivariate analysis of associations between underweight and potential risk factors (n=354∗)

Variable	Unadjusted†			Adjusted‡		
	OR	p value	CI	OR	p value	95% CI
Height (cm) (terciles) of mothers		0.0002		0.0016
<151	2.7		1.6–4.8	2.8		1.4–5.6
151–155	2.4		1.4–4.1	3.1		1.6–6.1
≥156	1		-	1		-
Level of schooling of mothers		0.041		0.055
None	2.2		1.2–3.9	2.7		1.2–6.1
Primary	2.0		1.0–4.0	2.8		1.2–6.9
Elementary	1.1		0.5–2.4	1.4		0.5–3.9
Secondary and more	1		-	1		-
Mother has professional occupation		0.0094		0.049
Yes	0.2		0.1–0.8	0.2		0.0–1.0
No	1		-	1		-
Father is head of household		0.088		0.14
Yes	0.6		0.4–1.1	0.6		0.3–1.2
No	1		-	1		-
Household water-quality index		0.05		0.014
Bad	2.1		0.8–5.4	5.6		1.4–21.9
Average	0.9		0.3–2.4	1.2		0.3–4.8
Good	1		-	1		-
Household income per person: ‘poverty gap’		0.024		0.23
<−100	3.0		1.3–6.7	2.7		0.8–8.5
−100 to +299	2.0		1.1–3.8	2.0		0.8–4.8
+300 and more	1		-	1		-
Birth-weight of children		<0.0001		0.0008
<2500 g	5.8		2.2–15.0	8.6		2.5–30.0
≥2,500 g	1		-	1		-
Child had diarrhoea in the last 2 weeks		0.084		0.011
Yes	1.7		0.9–3.3	3.1		1.3–7.5
No	1		-	1		-
Child was ever given formula regularly		0.089		0.018
Yes	1.5		0.9–2.5	2.2		1.1–4.1
No	1		-	1		-
Child eats from separate plate		0.043		0.034
Yes	1.6		1.0–2.6	2.0		1.0–3.6
No	1		-	1		-

∗ Based on the pairs for which values of all the retained variables were available;

† Univariate conditional logistic regression with dependent variable as in frst column;

‡ Multivariate conditional logistic regression: p values and odds ratios are adjusted for all variables in the table OR=Odds ratio; CI=Confdence interval

Although their distribution was markedly different between cases and controls, the variables—iron supplementation to mother and child health regularly monitored—were not included in the final multivariate model as they were more likely to be a consequence of an elevated risk of underweight (directly or not) than a potential cause.

After adjustment for one another in the multivariate model, most variables were still associated with underweight, except father being head of household and income of household expressed as ‘poverty gap’. For some variables, such as low birth-weight vs not, diarrhoea in the last two weeks vs none, or household water-quality index, the association with underweight was even stronger after adjustment. We specifically studied the quite drastic effect of the multivariate adjustment (unadjusted OR=5.8 vs adjusted OR=8.6) for the low birth-weight factor by running a series of analyses for which the adjustment was performed one variable at a time (data not shown). No single variable was found to explain by itself a sizeable part of this observed inverse confusion effect.

## DISCUSSION

The present study specifically considered a number of potential risk factors of underweight from the underlying and immediate categories and maternal/prenatal factors reported to be determinants of underweight of children in many developing countries (24,25). Among the underlying determinants, accessibility of health services was considered very good in the Omani context (12) and, thus, was not addressed.

The study had several limitations. The effect of age and sex on underweight could not be assessed due to the individual matching on these characteristics, but are well-documented in other contexts (26–28). Moreover, in 1995 and 1998, anthropometric status of Omani boys and girls was similar (6). The matching also suppressed the estimation of the effect of village of residence. Also a characteristic of the design is the choice of the underweight criteria index which mixes linear growth retardation (stunting defined as height-for-age index <−2 z-score) and/or wasting (weight-for-height index <−2 z-score). In our sample, 42% of the cases were stunted and 44% wasted, vs 10% and 6% respectively for the controls. These two forms of malnutrition usually have specific causes (29,30).

### Health of mother and prenatal factors

From our data, the factor that was more strongly associated with underweight was low birth-weight; as its effect was adjusted for all other covariates, including height of mother, it could be interpreted as an intermediate factor for other prenatal factors pertaining to maternal nutritional status. The effect on child nutritional status of the physical maternal and child prenatal characteristics, mediated through birth-weight, is well-documented (31) and was also recently reported in Oman (32). Maternal nutrition during pregnancy requires major attention, and iron supplementation for pregnant women, thought to improve birth outcome (33), was introduced in Oman in 1986; however, little is known about its compliance and impact. The observed higher prevalence of iron supplementation among mothers of cases was deemed more likely to be a consequence of an elevated risk of underweight (directly or not) than a potential cause. In our study, height of mother was also associated with underweight, independently of other covariates, including birth-weight and socioeconomic variables. Apart from the effect of possible genetic factors, this association with underweight may be related to the interpretation of adult height as a marker of social class distinctions (34) not entirely taken into account by the socioeconomic variables.

In fact, maternal height and low birth-weight are central in the intergenerational cycle of malnutrition (7) and can only be acted upon in the long term. The prevalence of low birth-weight in Oman remained around 8% from 1985 to 2002 (4), and in 1992, the average height of mothers was 155 cm (11). Height of mother was already reported as a possible cause of malnutrition through the intergenerational cycle when the prevalence of underweight was higher than expected from the development level (35).

### Environment, care, and health of children

In our study, the quality of household water was strongly associated with underweight as observed in other developing countries, e.g. Brazil (36). Historically, management of water resources is important in Oman where rain is scarce in most parts of the country: many villages or oasis still depend on the sophisticated ‘falaj’ underground irrigation system which dates back a thousand years. Also, contrary to nearly every other country, the coverage of water supply (37% in 1990 and 39% in 2000) in Oman is lower than the sanitation coverage (84% and 92% respectively) (37).

Diarrhoea was associated with underweight. However, it could be assumed that having diarrhoea could not only be a cause but also a consequence of being underweight. The occurrence of diarrhoea could be due to the use of bad-quality water, for example when using infant formula; however, because of adjustment for the quality of water in our study, the occurrence of diarrhoea could more likely be a marker of the ‘care for mothers and children’ category of underlying causes (38). Also in this category, feeding practices, such as regular use of infant formula or child-eating from a separate plate, were linked to underweight. This is in line with the findings that, in three of the regions sampled by our study, the variety, quality, and adequacy of complementary foods given to children are affected by lack of nutritional awareness among mothers in Oman (39).

### Socioeconomic environment

Schooling level of the mother and whether or not she was engaged in a professional occupation, that we found marginally associated to underweight, could partly act through prenatal factors as shown in another context in Congo (29), but may also be understood as a caring factor. In numerous developing countries, schooling of the mother and economic level of the household or income remained risk factors for child malnutrition (26,29,36,40,41). In Indonesia, another context of recent impressive public-health gain and decrease of underweight in children, maternal education, and household economic status have continued to be very strong predictors of the nutritional outcomes of children (42). Nevertheless, in our study, after adjustment, the household poverty gap was not associated with underweight. Assuming that income of the household could reflect household food security in Oman, the result underlines that this category of underlying causes is probably no longer relevant in the country. On the contrary, despite a good score on the life expectancy and gross domestic product GDP components of the human development indicators (1), Oman did not score so well on the education component due to an literacy rate of only 74.4% and a combined gross enrollment ratio for primary, secondary, and tertiary schools of 63%. There is also clearly a gender issue as the literacy rate is 65.4% for females as opposed to 82% for males (1).

In the Omani context, some determinants from the international conceptual model of the causes of malnutrition are probably no longer relevant, such as household food security or accessibility of health services. Some others are still pertinent, such as supply of safe water at the household level, and several factors relating to care for mothers and children, including education of mothers, prenatal nutritional status, and complementary child-feeding practices. Some of these factors, such as maternal nutrition during pregnancy, could be acted upon in the relatively short term. For some others, the solution is rather in the mid-term range, such as supply of safe water, education of mother, or even a distant future, such as height of mothers, which will at best improve only after several iterations of the intergenerational cycle. Further research is also needed to investigate further specific determinants of low birth-weight in the Omani context and try to disentangle emaciation and determinants of linear growth retardation. The research should not underestimate the importance of sociocultural factors, such as women's education and status.
